# Descriptive analysis of infections due to New Delhi metallo-β-lactamase-producing Enterobacterales in children at Red Cross War Memorial Children’s Hospital

**DOI:** 10.4102/sajid.v37i1.440

**Published:** 2022-07-27

**Authors:** Leonore Greybe, James J.C. Nuttall, Adrian J. Brink, Hafsah D. Tootla

**Affiliations:** 1 Pediatric Infectious Diseases Unit, Red Cross War Memorial Children’s Hospital, Cape Town, South Africa; 2 Department of Paediatrics and Child Health, University of Cape Town, Cape Town, South Africa; 3 Microbiology Laboratory, National Health Laboratory Service, Groote Schuur Hospital and Red Cross War Memorial Children’s Hospital, Cape Town, South Africa; 4 Division of Medical Microbiology, Department of Pathology, Faculty of Health Sciences, University of Cape Town, South Africa

**Keywords:** paediatrics, *Serratia marcescens*, Enterobacterales, antibiotic resistance, carbapenem resistance, New Delhi metallo-β-lactamase, carbapenemases

## Abstract

The increased incidence and absence of antibiotic treatment options for New Delhi metallo-β-lactamase (NDM)-producing carbapenem-resistant Enterobacterales (CRE) infection are concerning. Recent reports have highlighted NDM-producing *Serratia marcescens*, as a specific concern, as it is an organism which is intrinsically resistant to colistin. In this study, a descriptive analysis of NDM-producing CRE infections was performed at the Red Cross War Memorial Children’s Hospital.

## Introduction

Carbapenem resistance in Enterobacterales is most commonly mediated by the production of carbapenemases that hydrolyse almost all β-lactams (BLs), including the carbapenems that are considered the last-line and therapeutic choice for severe infections caused by extended spectrum β-lactamase (ESBL)-producing Enterobacterales.^1,2^ Carbapenemases can be classified into those with a serine residue (class A and D) and those with zinc (class B or metallo-β-lactamase [MBL] enzymes) at their active site. New Delhi metallo-β-lactamase (NDM) is a class B carbapenemase.^3^

A recent publication on the epidemiology of carbapenem-resistant Enterobacterales (CRE) bacteraemia in South African tertiary hospitals included a significant number of children and adolescents (485/1293, 38%) and predominantly identified the class D Oxacillinase-48 (OXA-48) carbapenemase and its variants (52%), followed by the class B NDM (34%) carbapenemase. The most common bacteria identified in this study were *Klebsiella pneumoniae, Enterobacter cloacae* and *Serratia marcescens*.^4^ Furthermore, in 2017, Moodley et al. raised concern about the emergence of CRE *S. marcescens* harbouring the *NDM-1* gene, where 10/63 (16%) *S. marcescens* isolates from blood cultures across the KwaZulu-Natal region carried this gene.^5^ This high prevalence of NDM-producing CRE, and specifically those caused by *S. marcescens*, is a significant public health concern as *S. marcescens* is intrinsically resistant to colistin, the antibiotic of choice for CRE infections in low-income settings, with no alternative directed treatment available.^6^

Aztreonam-avibactam is being investigated in clinical trials for the treatment of NDM-producing CRE.^3^ In the interim, cefiderocol or a combination therapy with ceftazidime-avibactam (CA) and aztreonam (AT) has shown promising results for the treatment of NDM CRE infection. Cost, registration status with the South African Health Products Regulatory Authority, limited access to the antibiotics and susceptibility testing, and uncertainty of dosage and duration of therapy in children are some of the challenges.^2,7,8^ Similar issues will be expected with aztreonam-avibactam when it becomes available for clinical use.

Due to an increase in isolation of NDM-producing CRE at the health care institution, a retrospective descriptive analysis of these isolates was performed and includes epidemiological and microbiological characteristics, as well as management and outcome measures.

## Methods

The National Health Laboratory Service database was used to identify patients with carbapenem non-susceptible Enterobacterales (minimum inhibitory concentrations [MICs] ≥ 2 µg/mL for meropenem or imipenem, and ≥ 1 µg/mL for ertapenem) using the VITEK 2^®^ (bioMérieux, Marcy-l’Étoile, France) automated susceptibility testing system, from clinical samples (excluding rectal swabs/stool surveillance samples) collected at RCWMCH during 01 October 2015 – 28 February 2021. Patients were included in the study if an NDM carbapenemase was detected from these isolates either via PCR (performed at the National Institute for Communicable Diseases, Johannesburg, South Africa) prior to 2019, or phenotypically thereafter using the RESIST-4 O.K.N.V^®^ (Coris BioConcept, Gembloux, Belgium) lateral flow assay. Carbapenem ETEST (bioMérieux) MICs were performed on selected isolates. Colistin susceptibility was performed on selected isolates using broth microdilution (BMD) (performed at Charlotte Maxeke Infection Control Laboratory, Johannesburg, South Africa). The records of these children were reviewed retrospectively to identify demographics, risk factors, microbiological characteristics, management interventions and outcomes. A descriptive analysis of the data was performed using Microsoft Excel (version 2111).

### Ethical considerations

Ethics approval was obtained from the University of Cape Town Human Research Ethics Committee. (HREC Ref: 238/2021) The use of archived data justified a waiver of informed consent.

## Results

Twenty-six NDM-producing CRE isolates from 22 children were included in the study.

### Demographics

Most children included were female (59%). The median age at the time of culture was 18 (interquartile range [IQR]: 5–75) months. The median time in hospital prior to isolation of an NDM-producing CRE was 19 (IQR: 4–34) days, with four children (18%) infected or colonised at admission. The median overall length of stay in hospital was 37 (IQR: 15–59) days. The median time from NDM-producing CRE isolation to discharge or death was 14 (IQR: 5–25) and 12 (IQR: 4–44) days, respectively.

### Risk factors

**FIGURE 1:**
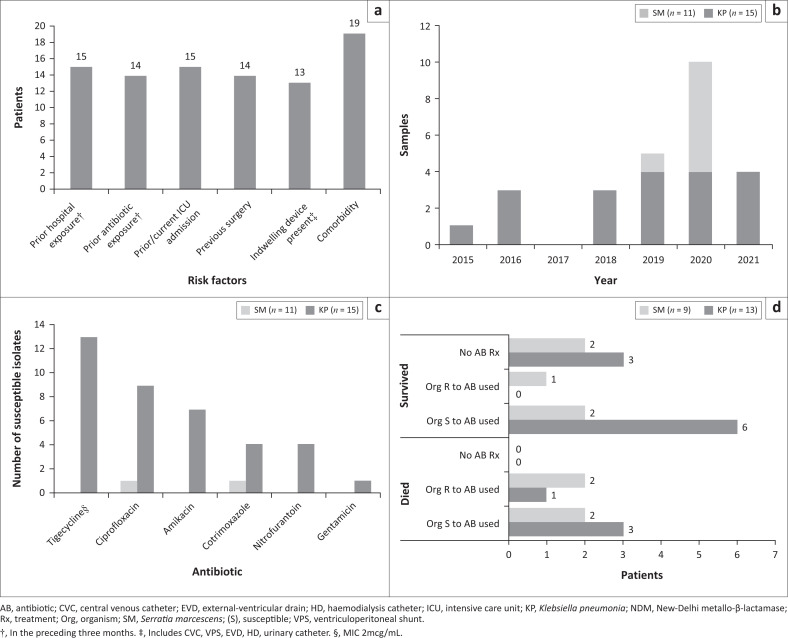
Graphic representation of (a) risk factors for infection with NDM-producing Enterobacterales, (b) the number of NDM-producing Enterobacterales identified during the study period by organism, (c) antibiogram of NDM-producing Enterobacterales (number of susceptible isolates to currently available antibiotics), and (d) outcome stratified by organism, antibiotic susceptibility and antibiotic treatment received.

All patients had at least one known risk factor for CRE infection,^6,9^ with three or more risk factors present in 18/22 (82%) patients. The risk factors included comorbidity (19/22, 86%) (Table 1), exposure to a health care facility in the preceding 3 months (15/22, 68%), prior or current intensive care unit (ICU) admission (15/22, 68%), previous surgery (14/22, 63%), presence of an indwelling device (13/22, 59%) and antibiotic exposure in the preceding three months (14/22, 64%) (Figure 1a).

**TABLE 1:** Clinical characteristics, management and outcome of children with NDM-producing Enterobacterales.

Patient	Organism(s)	Sample(s)	Clinical information	Management	Outcome
1	KP	RESP	8-month-old, with DiGeorge syndrome, vocal cord paralysis, tracheostomy and VAP.	Cotrimoxazole (S)	Survived
2	KP	RESP	129-month-old with chronic liver failure secondary to biliary atresia, failed Kasai procedure and VAP.	Ciprofloxacin (S)	Survived
3	KP	URINE	29-month-old, with encephalitis and subsequent catheter-associated UTI.	Urinary catheter removed Meropenem (No E-test)	Survived
4	KP	URINE	78-month-old, with anorectal malformation and previous urethroplasty with catheter-associated UTI.	Urinary catheter removed Ciprofloxacin (S)	Survived
6	KP	URINE	75-month-old, with newly diagnosed HIV, fever and no UTI symptoms.	None Categorised as a contaminant. (No leukocytes on microscopy)	Survived
7	SA, KP KP	TISS URINE	4-month-old, with hydrocephalus, and local wound sepsis at VPS insertion site and UTI.	VPS exchanged for EVD Urinary catheter removed Ceftriaxone (S) and Ciprofloxacin (S)	Survived
8	KP KP	SWAB TISS	15-month-old, with tracheoesophageal atresia with a colonic interposition graft. Samples taken from morphologically normal colon graft tissue during endoscopy.	None Categorised as a coloniser of colonic tissue	Survived
9	KP	SWAB	45-month-old, with nephrotic syndrome on haemodialysis with HD catheter infection.	HD catheter removed Ciprofloxacin (S)	Survived
10	KP	BC	6-month-old with no comorbidities and community-acquired pneumonia.	Ampicillin/Gentamicin (good clinical response) Organism categorised as a contaminant	Survived
11	KP	URINE	36-month-old, with 65% hot water burns, failed allografts and catheter-associated UTI. Sudden death 5 days later.	Amikacin (S)	Died
12	KP	BC	133-month-old, receiving chemotherapy for Langerhans cell histiocytosis with febrile neutropenia.	Meropenem (MIC ≥ 32 μg/mL) Colistin (MIC = 1 μg/mL) Cotrimoxazole (R)	Died
13	KP	BC	1-month-old baby with bowel obstruction and NEC.	Source control surgery Meropenem (MIC = 4 μg/mL) Ciprofloxacin (S) Vancomycin	Died
14	KP, MTB, HS	TISS (Post-mortem)	78-month-old, with fatal sepsis on admission.	Ceftriaxone (treated empirically as a community acquired infection) (R)	Died
15	SM	RESP	2-day-old with meconium peritonitis and suspected cystic fibrosis, with VAP.	Mero (MIC ≥ 32 μg/mL)	Survived
16	SM	URINE	18-month-old with newly diagnosed rhabdomyosarcoma and catheter-associated UTI.	Ciprofloxacin (S)	Survived
17	SM	CVC	2-day-old recovering from gastroschisis surgery. Asymptomatic.	None Line removed	Survived
18	SM	SWAB	Healthy 84-month-old child with preauricular sinus abscess.	Topical chloramphenicol (susceptibility not performed)	Survived
19	SM, EF	SWAB	1-day-old premature neonate with NEC.	Surgery Imipenem (MIC ≥ 32 μg/mL) Colistin (R)	Died
20	SM	BC	75-month-old, with Fanconi syndrome, malignant transformation and failed bone marrow transplant. Severe immunosuppression with sepsis.	Meropenem (MIC = 0.25 μg/mL) Ciprofloxacin (S)	Died
21	SM SM	BC SWAB	2-day-old with surgical site infection after surgery for congenital heart disease.	Meropenem (MIC ≥ 32 μg/mL)	Died
22	SM SM	BC SWAB	168-month-old with renal transplant rejection and necrotizing pancreatitis.	Meropenem (MIC ≥ 32 μg/mL) Cotrimoxazole (S)	Died

BC, blood culture; CVC, central venous catheter tip; EF, *Enterococcus faecalis*; EVD, external-ventricular drain; HD, haemodialysis catheter; HS, *Herpes simplex*; HIV, human immunodeficiency virus; KP, *Klebsiella pneumoniae*; MIC, minimum inhibitory concentration; MTB, *Mycobacterium tuberculosis*; NEC, necrotizing enterocolitis; (R), resistant; RESP, respiratory sample; SWAB, pus swab; SA, *Staphylococcus aureus*; SM, *Serratia marcescens*; (S), susceptible; TISS, tissue; UTI, urinary tract infection; VAP, ventilator- associated pneumonia; VPS, ventriculoperitoneal shunt.

### Microbiology

New Delhi metallo-β-lactamase-producing CRE were isolated from blood culture (7/26, 27%), urine (6/26, 23%), pus (6/26, 23%), respiratory samples (3/26, 12%), tissue (3/26, 12%), and a catheter tip (1/26, 4%). Four children (18%) had NDM-producing CRE isolated from two different sites, including blood culture and pus,^2^ pus swab and tissue,^1^ and tissue and urine.^1^

Only NDM-producing *K. pneumoniae* (15/26, 58%) and *S. marcescen*s (11/26, 42%) were isolated. As depicted in Figure 1b, *K. pneumoniae* was isolated from one (4%) sample in 2015, three (12%) in 2016, none in 2017, three (12%) in 2018 and 2019, five (19%) in 2020 and no samples in 2021. NDM-producing *S. marcescens* was first identified in 2019 from a single (4%) sample. Thereafter, *S. marcescens* was identified from six (23%) samples in 2020, and all four (15%) CRE samples in 2021.

Only two isolates (8%), both *K. pneumoniae* from different children, had dual carbapenemases present with Oxa-48 and variants also detected.

Out of the 15 *K. pneumoniae* isolates, 2 (2/15, 13%) were pan drug-resistant to all antibiotics tested, including colistin resistance in the one isolated and tested. The remaining isolates were susceptible to tigecycline (13/15, 87%), ciprofloxacin (9/15, 60%), amikacin (7/15, 47%), trimethoprim-sulfamethoxazole (4/15, 27%) and nitrofurantoin [4/15, (27%), but only one (7%) of these was isolated from urine and gentamicin (1/15, 7%) (Figure 1c). Colistin BMD was performed on two isolates, and both had MICs ≤ 2 µg/mL. Nine isolates had carbapenem E-tests performed and five of these (56%) had meropenem MICs ≤ 8 µg/mL.

Out of the 11 *S. marcescen*s isolates, 9 (82%) were pan drug-resistant. One isolate (9%) was susceptible to trimethoprim-sulfamethoxazole and one (9%) susceptible to ciprofloxacin. Ten isolates had carbapenem E-tests performed and eight (80%) of these had meropenem MICs ≥ 32 µg/mL.

### Management and outcome

The clinical characteristics, management and outcome of all patients are summarised in Table 1. In total, there were 17/22 (77%) patients who had clinically significant infection and isolated *K. pneumoniae* (10/13, 77%) and *S. marcescens* (7/9, 78%) from their samples, respectively. Nine of the patients (6/9, 67% *K. pneumoniae*; 3/9, 33% *S. marcescens*) who were diagnosed with ventilator-associated pneumonia, urinary tract infection, local wound infection or intravascular catheter-related infection were successfully treated. Of these, five (5/6, 83%) *K. pneumoniae* isolates and two (2/3, 67%) *S. marcescens* isolates demonstrated *in vitro* susceptibility to at least one antibiotic used for treatment. Eight patients (8/17, 47%), four (4/8, 50%) *K. pneumoniae* and four (4/8, 50%) *S. marcescens*, died. The patients had blood stream infection, intra-abdominal infection, surgical wound infection or urinary tract infection. One (1/4, 25%) with *K. pneumoniae* and three (3/4, 75%) with *S. marcescens* were treated with antibiotics that did not demonstrate any *in vitro* susceptibility (Table 1).

Five (5/22, 23%) patients, three (3/13, 23%) *K. pneumoniae* and two (2/9, 22%) *S. marcescens*, did not have clinically significant infection. These organisms were classified as contaminants or colonisers and were not treated with antibiotics directed at the CRE organism.

## Discussion

The increase in CRE infections, and in particular NDM-producing *S. marcescens*, has been identified as a public health concern with the potential for outbreaks and being untreatable.^9,10^ In keeping with this trend, the authors noted increasing numbers of NDM-producing CRE and specifically NDM-producing *S. marcescens* from clinical samples at the healthcare institution. A total of 81% of children who isolated NDM CRE had ≥ 3 risk factors present, with the majority being the presence of comorbidities, prior hospitalisation and antibiotic exposure in the preceding three months, and ICU admission, highlighting the vulnerable population that NDM CRE affects. New Delhi metallo-β-lactamase was first identified in *K. pneumoniae,* and then alarmingly in *S. marcescens,* which has subsequently become the predominant NDM-producing organism at the institution.

Resistance to antibiotics was higher in *S. marcescens* than in *K. pneumoniae* with the majority being pan drug-resistant (9/11, 82%) and having high meropenem MICs (≥ 32 µg/mL) (8/10, 80%), when compared to the lower proportion of pan drug-resistant *K. pneumoniae* isolates (2/15, 13%) and those with high meropenem MICs (5/9, 56%). However, it is also important to be aware that BL susceptibility testing of MBL-producing organisms is influenced by the presence of zinc in the testing medium, which if depleted may render lower MICs or false susceptibility to BL antibiotics, making interpretation additionally challenging.^3^ Further complicating this are NDM CRE case reports and simulated human models describing successful clinical outcomes and reduction in bacterial loads, respectively, using carbapenems, despite high MICs.^11,12^

Treatment of CRE infections in children, and those caused by NDM-producers, is confounded by a paucity of data-driven evidence and availability of effective antibiotics. Treatment is usually individualised based on *in vitro* susceptibility profiles and pharmacokinetics of available but less optimal antibiotics, such as combination or monotherapy with carbapenems, aminoglycosides, fluoroquinolones, polymyxins, and tigecycline. These antibiotics are used with various degrees of success depending on the severity and site of infection, and whether source control can be attained. Outcomes in children vary, with descriptions of lower mortality in those with NDM infection when compared to non-NDM infection being reported,^12^ but this may evolve as the incidence of NDM CRE infection increases. Overall paediatric CRE infection outcomes are often poor in severe illness despite individualised therapy^6^ and are unlikely to change until new β-lactamase inhibitor combinations (BLICs), such as aztreonam-avibactam, become available. Based on limited evidence from using the combination of CA and AT for the management of NDM CRE infection, the authors have advocated for its use in selected patients with serious NDM CRE infection and include those with invasive infection with high MIC’s to meropenem and non-susceptibility (intrinsic in the case of *S. marcescens*) or treatment failure with colistin.

Antimicrobial Stewardship Programmes through institutional and individualised guidance play a major role in the management of these complex infections and perhaps, more importantly, avoiding these infections by limiting selection pressure through the judicious use of broad-spectrum antibiotics. The use of CRE surveillance data as well as technological advances in the identification of (multi-drug resistant) micro-organisms can also limit unnecessary prolonged exposure to broad spectrum antibiotics.^13^

Finally, it is critical to always reinforce compliance with infection prevention and control such as hand hygiene, barrier nursing, and enhanced environmental cleaning measures, to limit the spread of these organisms within institutions, as developing new antibiotics only partially address the problem of multidrug-resistant organisms and is neither an immediate nor sustainable solution to the crisis.

Limitations of this study analysis include not estimating the longitudinal prevalence rate of CRE at the Red Cross War Memorial Children’s Hospital. The impact of delays in appropriate directed therapy, due to limited access to expedited pheno- and genotypic susceptibility testing, could not be analysed. The differences in severity of illness, which is an important outcome determinant, were also not compared. In addition, as whole-genome sequencing of CRE was not performed, resistome and virolome determinants, phylogenetic and plasmid analysis, and thus transmission dynamics at the institution is unknown. This may be important to elucidate the underlying reasons for the replacement of *K. pneumoniae* by NDM *S. marcescens* and its current dominance. Going forward, such data are pivotal, not only to inform data-driven patient management, but also diagnostic and antibiotic stewardship and infection prevention strategies.

## Conclusion

Reports of NDM CRE are increasing, with NDM-producing *S. marcescens* specifically being identified as a serious public health threat. This study highlights the paucity of antibiotics available to treat these infections, especially those caused by NDM *S. marcescens*, which appear to be more resistant to the backbone treatment options for CRE infection.
